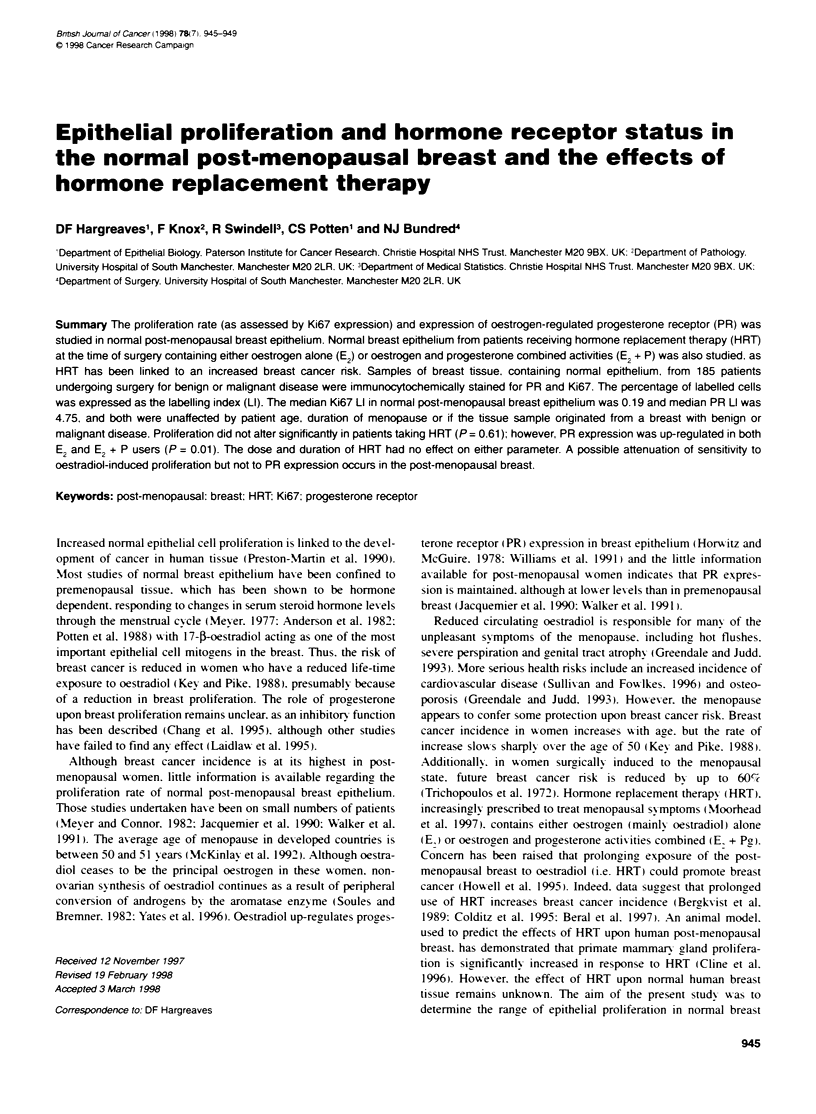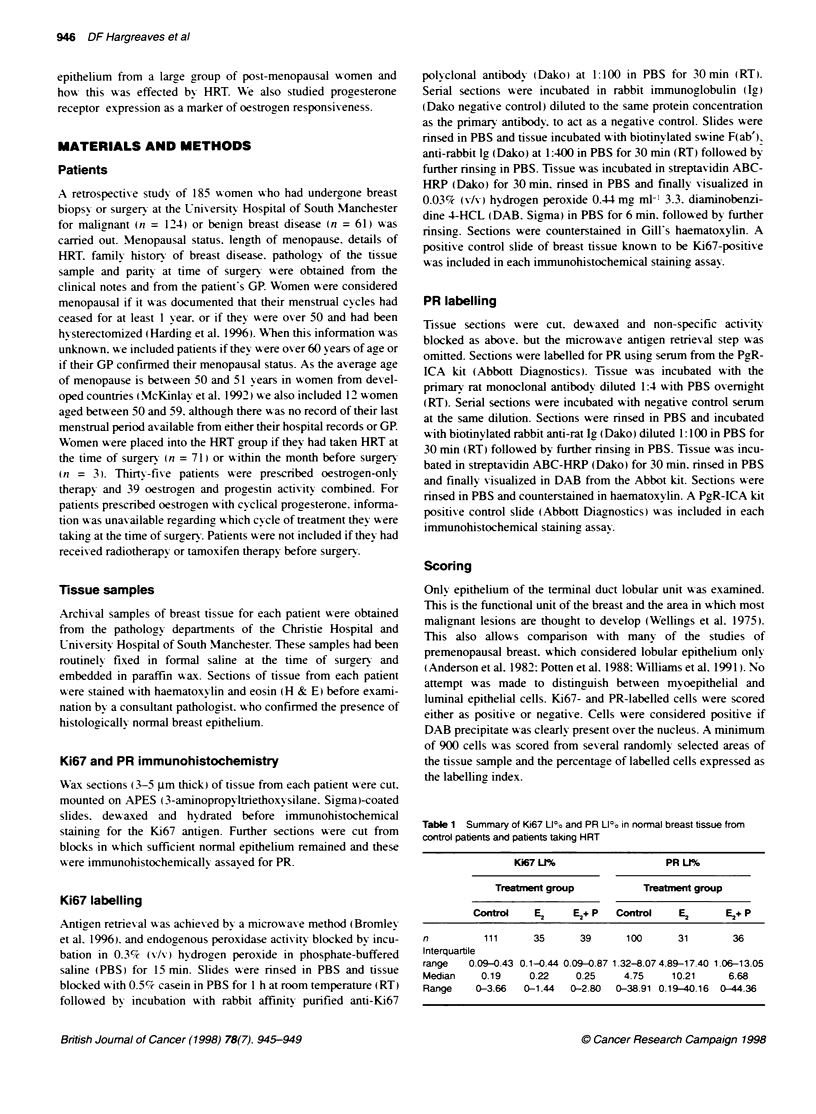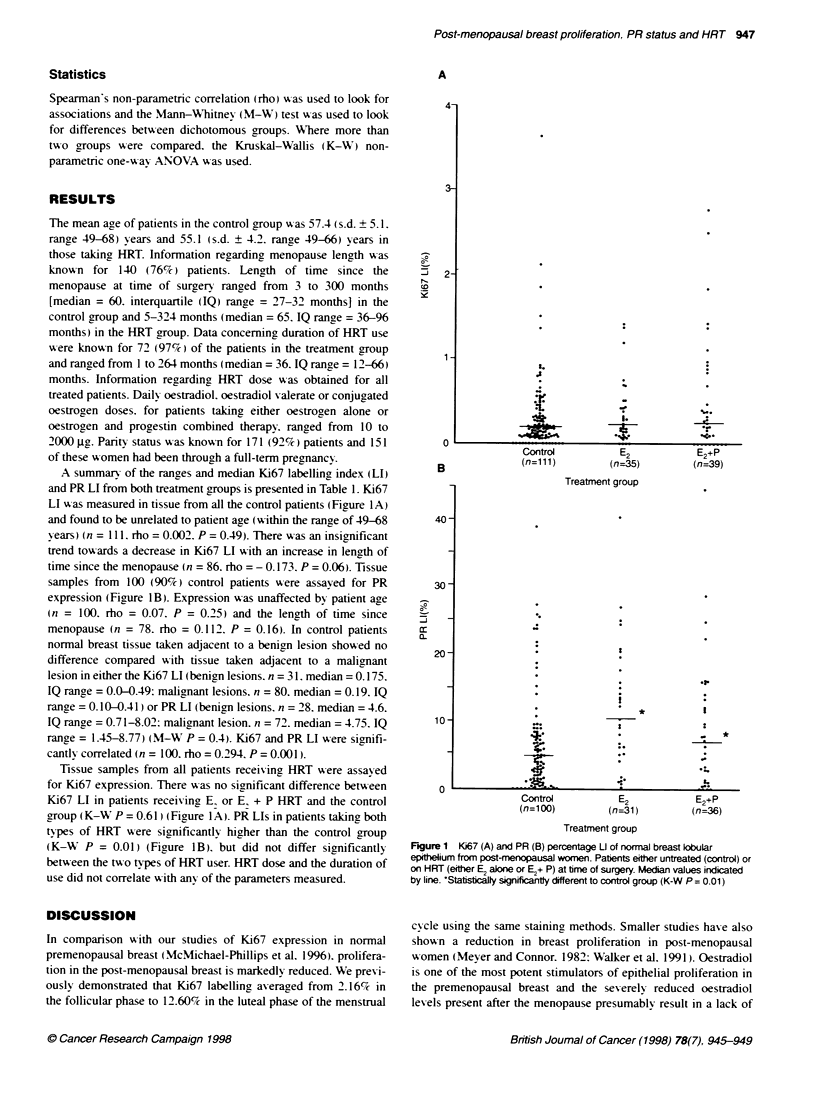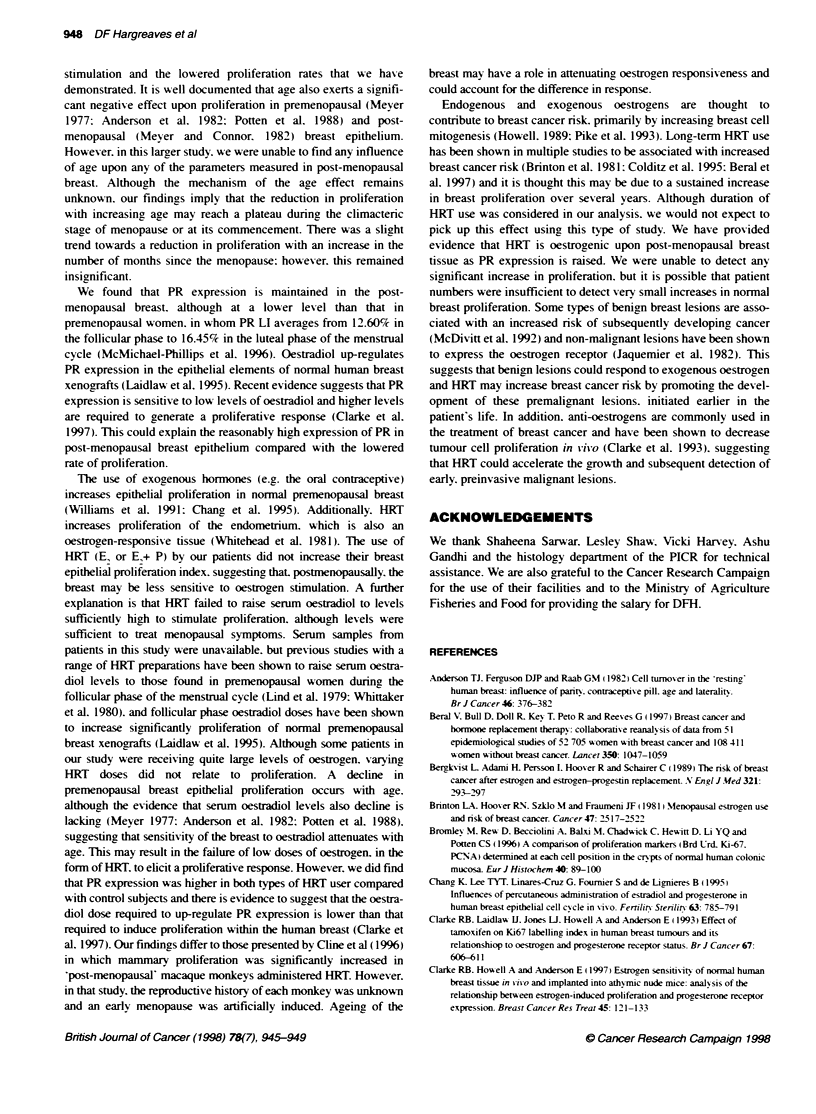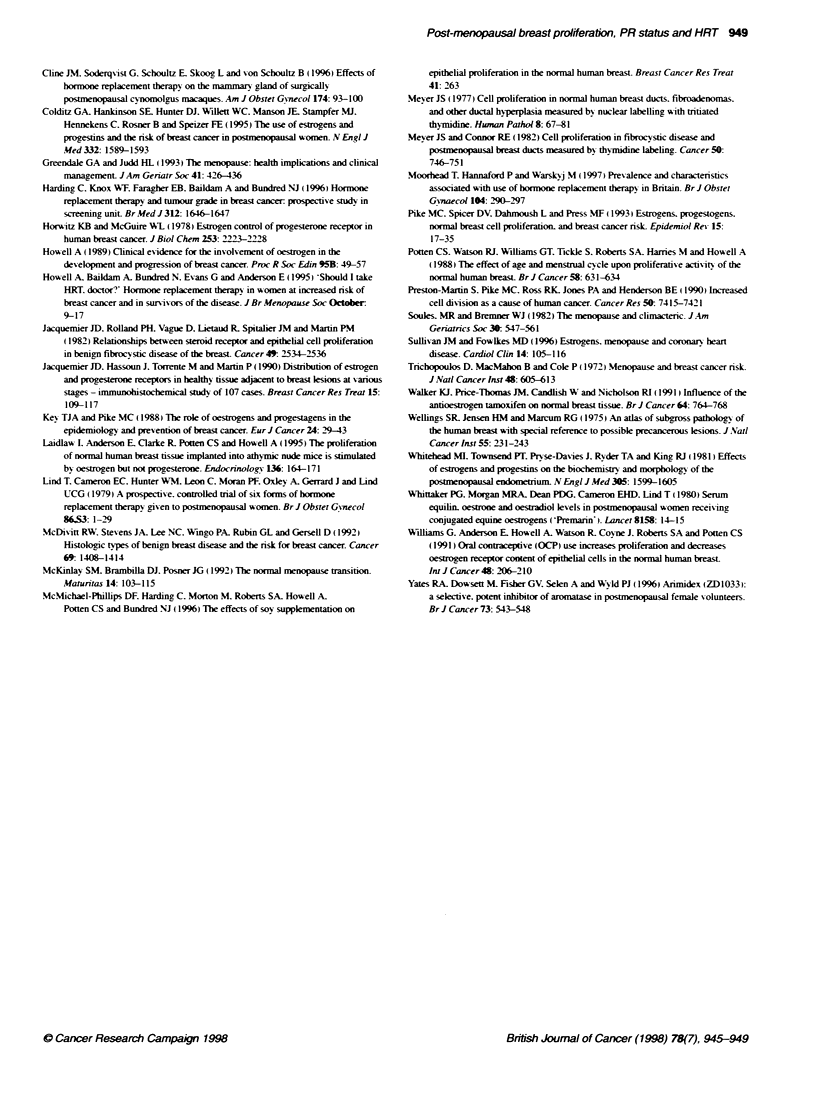# Epithelial proliferation and hormone receptor status in the normal post-menopausal breast and the effects of hormone replacement therapy.

**DOI:** 10.1038/bjc.1998.606

**Published:** 1998-10

**Authors:** D. F. Hargreaves, F. Knox, R. Swindell, C. S. Potten, N. J. Bundred

**Affiliations:** Department of Epithelial Biology, Paterson Institute for Cancer Research, Christie Hospital NHS Trust, Manchester, UK.

## Abstract

The proliferation rate (as assessed by Ki67 expression) and expression of oestrogen-regulated progesterone receptor (PR) was studied in normal post-menopausal breast epithelium. Normal breast epithelium from patients receiving hormone replacement therapy (HRT) at the time of surgery containing either oestrogen alone (E2) or oestrogen and progesterone combined activities (E2 + P) was also studied, as HRT has been linked to an increased breast cancer risk. Samples of breast tissue, containing normal epithelium, from 185 patients undergoing surgery for benign or malignant disease were immunocytochemically stained for PR and Ki67. The percentage of labelled cells was expressed as the labelling index (LI). The median Ki67 LI in normal post-menopausal breast epithelium was 0.19 and median PR LI was 4.75, and both were unaffected by patient age, duration of menopause or if the tissue sample originated from a breast with benign or malignant disease. Proliferation did not alter significantly in patients taking HRT (P = 0.61); however, PR expression was up-regulated in both E2 and E2 + P users (P = 0.01). The dose and duration of HRT had no effect on either parameter. A possible attenuation of sensitivity to oestradiol-induced proliferation but not to PR expression occurs in the post-menopausal breast.


British Journal of Cancer (1998) 78(7), 945–949
© 1998 Cancer Research Campaign

### Epithelial proliferation and hormone receptor status in the normal post-menopausal breast and the effects of hormone replacement therapy

**DF Hargreaves[1], F Knox[2], R Swindell[3], CS Potten[1] and NJ Bundred[4]**

[1]Department of Epithelial Biology, Paterson Institute for Cancer Research, Christie Hospital NHS Trust, Manchester M20 9BX, UK; [2]Department of Pathology, University Hospital of South Manchester, Manchester M20 2LR, UK; [3]Department of Medical Statistics, Christie Hospital NHS Trust, Manchester M20 9BX, UK; [4]Department of Surgery, University Hospital of South Manchester, Manchester M20 2LR, UK

**Summary** The proliferation rate (as assessed by Ki67 expression) and expression of oestrogen-regulated progesterone receptor (PR) was studied in normal post-menopausal breast epithelium. Normal breast epithelium from patients receiving hormone replacement therapy (HRT) at the time of surgery containing either oestrogen alone ($E_2$) or oestrogen and progesterone combined activities ($E_2$ + P) was also studied, as HRT has been linked to an increased breast cancer risk. Samples of breast tissue, containing normal epithelium, from 185 patients undergoing surgery for benign or malignant disease were immunocytochemically stained for PR and Ki67. The percentage of labelled cells was expressed as the labelling index (LI). The median Ki67 LI in normal post-menopausal breast epithelium was 0.19 and median PR LI was 4.75, and both were unaffected by patient age, duration of menopause or if the tissue sample originated from a breast with benign or malignant disease. Proliferation did not alter significantly in patients taking HRT ($P$ = 0.61); however, PR expression was up-regulated in both $E_2$ and $E_2$ + P users ($P$ = 0.01). The dose and duration of HRT had no effect on either parameter. A possible attenuation of sensitivity to oestradiol-induced proliferation but not to PR expression occurs in the post-menopausal breast.

**Keywords:** post-menopausal; breast; HRT; Ki67; progesterone receptor

Increased normal epithelial cell proliferation is linked to the development of cancer in human tissue (Preston-Martin et al, 1990). Most studies of normal breast epithelium have been confined to premenopausal tissue, which has been shown to be hormone dependent, responding to changes in serum steroid hormone levels through the menstrual cycle (Meyer, 1977; Anderson et al, 1982; Potten et al, 1988) with 17-β-oestradiol acting as one of the most important epithelial cell mitogens in the breast. Thus, the risk of breast cancer is reduced in women who have a reduced life-time exposure to oestradiol (Key and Pike, 1988), presumably because of a reduction in breast proliferation. The role of progesterone upon breast proliferation remains unclear, as an inhibitory function has been described (Chang et al, 1995), although other studies have failed to find any effect (Laidlaw et al, 1995).

Although breast cancer incidence is at its highest in post-menopausal women, little information is available regarding the proliferation rate of normal post-menopausal breast epithelium. Those studies undertaken have been on small numbers of patients (Meyer and Connor, 1982; Jacquemier et al, 1990; Walker et al, 1991). The average age of menopause in developed countries is between 50 and 51 years (McKinlay et al, 1992). Although oestradiol ceases to be the principal oestrogen in these women, non-ovarian synthesis of oestradiol continues as a result of peripheral conversion of androgens by the aromatase enzyme (Soules and Bremner, 1982; Yates et al, 1996). Oestradiol up-regulates progesterone receptor (PR) expression in breast epithelium (Horwitz and McGuire, 1978; Williams et al, 1991) and the little information available for post-menopausal women indicates that PR expression is maintained, although at lower levels than in premenopausal breast (Jacquemier et al, 1990; Walker et al, 1991).

Reduced circulating oestradiol is responsible for many of the unpleasant symptoms of the menopause, including hot flushes, severe perspiration and genital tract atrophy (Greendale and Judd, 1993). More serious health risks include an increased incidence of cardiovascular disease (Sullivan and Fowlkes, 1996) and osteoporosis (Greendale and Judd, 1993). However, the menopause appears to confer some protection upon breast cancer risk. Breast cancer incidence in women increases with age, but the rate of increase slows sharply over the age of 50 (Key and Pike, 1988). Additionally, in women surgically induced to the menopausal state, future breast cancer risk is reduced by up to 60% (Trichopoulos et al, 1972). Hormone replacement therapy (HRT), increasingly prescribed to treat menopausal symptoms (Moorhead et al, 1997), contains either oestrogen (mainly oestradiol) alone ($E_2$) or oestrogen and progesterone activities combined ($E_2$ + Pg). Concern has been raised that prolonging exposure of the post-menopausal breast to oestradiol (i.e. HRT) could promote breast cancer (Howell et al, 1995). Indeed, data suggest that prolonged use of HRT increases breast cancer incidence (Bergkvist et al, 1989; Colditz et al, 1995; Beral et al, 1997). An animal model, used to predict the effects of HRT upon human post-menopausal breast, has demonstrated that primate mammary gland proliferation is significantly increased in response to HRT (Cline et al, 1996). However, the effect of HRT upon normal human breast tissue remains unknown. The aim of the present study was to determine the range of epithelial proliferation in normal breast

*Received 12 November 1997*
*Revised 19 February 1998*
*Accepted 3 March 1998*

*Correspondence to:* DF Hargreaves epithelium from a large group of post-menopausal women and how this was effected by HRT. We also studied progesterone receptor expression as a marker of oestrogen responsiveness.

### MATERIALS AND METHODS

### Patients

A retrospective study of 185 women who had undergone breast biopsy or surgery at the University Hospital of South Manchester for malignant ($n$ = 124) or benign breast disease ($n$ = 61) was carried out. Menopausal status, length of menopause, details of HRT, family history of breast disease, pathology of the tissue sample and parity at time of surgery were obtained from the clinical notes and from the patient's GP. Women were considered menopausal if it was documented that their menstrual cycles had ceased for at least 1 year, or if they were over 50 and had been hysterectomized (Harding et al. 1996). When this information was unknown, we included patients if they were over 60 years of age or if their GP confirmed their menopausal status. As the average age of menopause is between 50 and 51 years in women from developed countries (McKinlay et al. 1992) we also included 12 women aged between 50 and 59, although there was no record of their last menstrual period available from either their hospital records or GP. Women were placed into the HRT group if they had taken HRT at the time of surgery ($n$ = 71) or within the month before surgery ($n$ = 3). Thirty-five patients were prescribed oestrogen-only therapy and 39 oestrogen and progestin activity combined. For patients prescribed oestrogen with cyclical progesterone, information was unavailable regarding which cycle of treatment they were taking at the time of surgery. Patients were not included if they had received radiotherapy or tamoxifen therapy before surgery.

### Tissue samples

Archival samples of breast tissue for each patient were obtained from the pathology departments of the Christie Hospital and University Hospital of South Manchester. These samples had been routinely fixed in formal saline at the time of surgery and embedded in paraffin wax. Sections of tissue from each patient were stained with haematoxylin and eosin (H & E) before examination by a consultant pathologist, who confirmed the presence of histologically normal breast epithelium.

### Ki67 and PR immunohistochemistry

Wax sections (3–5 μm thick) of tissue from each patient were cut, mounted on APES (3-aminopropyltriethoxysilane, Sigma)-coated slides, dewaxed and hydrated before immunohistochemical staining for the Ki67 antigen. Further sections were cut from blocks in which sufficient normal epithelium remained and these were immunohistochemically assayed for PR.

### Ki67 labelling

Antigen retrieval was achieved by a microwave method (Bromley et al. 1996), and endogenous peroxidase activity blocked by incubation in 0.3% (v/v) hydrogen peroxide in phosphate-buffered saline (PBS) for 15 min. Slides were rinsed in PBS and tissue blocked with 0.5% casein in PBS for 1 h at room temperature (RT) followed by incubation with rabbit affinity purified anti-Ki67 polyclonal antibody (Dako) at 1:100 in PBS for 30 min (RT). Serial sections were incubated in rabbit immunoglobulin (Ig) (Dako negative control) diluted to the same protein concentration as the primary antibody, to act as a negative control. Slides were rinsed in PBS and tissue incubated with biotinylated swine $F(ab')_2$ anti-rabbit Ig (Dako) at 1:400 in PBS for 30 min (RT) followed by further rinsing in PBS. Tissue was incubated in streptavidin ABC-HRP (Dako) for 30 min, rinsed in PBS and finally visualized in 0.03% (v/v) hydrogen peroxide 0.44 mg $ml^{-1}$ 3,3, diaminobenzidine 4-HCL (DAB, Sigma) in PBS for 6 min, followed by further rinsing. Sections were counterstained in Gill's haematoxylin. A positive control slide of breast tissue known to be Ki67-positive was included in each immunohistochemical staining assay.

### PR labelling

Tissue sections were cut, dewaxed and non-specific activity blocked as above, but the microwave antigen retrieval step was omitted. Sections were labelled for PR using serum from the PgR-ICA kit (Abbott Diagnostics). Tissue was incubated with the primary rat monoclonal antibody diluted 1:4 with PBS overnight (RT). Serial sections were incubated with negative control serum at the same dilution. Sections were rinsed in PBS and incubated with biotinylated rabbit anti-rat Ig (Dako) diluted 1:100 in PBS for 30 min (RT) followed by further rinsing in PBS. Tissue was incubated in streptavidin ABC-HRP (Dako) for 30 min, rinsed in PBS and finally visualized in DAB from the Abbot kit. Sections were rinsed in PBS and counterstained in haematoxylin. A PgR-ICA kit positive control slide (Abbott Diagnostics) was included in each immunohistochemical staining assay.

### Scoring

Only epithelium of the terminal duct lobular unit was examined. This is the functional unit of the breast and the area in which most malignant lesions are thought to develop (Wellings et al. 1975). This also allows comparison with many of the studies of premenopausal breast, which considered lobular epithelium only (Anderson et al. 1982; Potten et al. 1988; Williams et al. 1991). No attempt was made to distinguish between myoepithelial and luminal epithelial cells. Ki67- and PR-labelled cells were scored either as positive or negative. Cells were considered positive if DAB precipitate was clearly present over the nucleus. A minimum of 900 cells was scored from several randomly selected areas of the tissue sample and the percentage of labelled cells expressed as the labelling index.

**Table 1** Summary of Ki67 LI% and PR LI% in normal breast tissue from control patients and patients taking HRT

| | Ki67 LI% | | | PR LI% | | |
|---|---|---|---|---|---|---|
| | Treatment group | | | Treatment group | | |
| | Control | $E_2$ | $E_2$+ P | Control | $E_2$ | $E_2$+ P |
| $n$ | 111 | 35 | 39 | 100 | 31 | 36 |
| Interquartile range | 0.09–0.43 | 0.1–0.44 | 0.09–0.87 | 1.32–8.07 | 4.89–17.40 | 1.06–13.05 |
| Median | 0.19 | 0.22 | 0.25 | 4.75 | 10.21 | 6.68 |
| Range | 0–3.66 | 0–1.44 | 0–2.80 | 0–38.91 | 0.19–40.16 | 0–44.36 |

 © Cancer Research Campaign 1998

### Statistics

Spearman's non-parametric correlation (rho) was used to look for associations and the Mann–Whitney (M–W) test was used to look for differences between dichotomous groups. Where more than two groups were compared, the Kruskal–Wallis (K–W) non-parametric one-way ANOVA was used.

### RESULTS

The mean age of patients in the control group was 57.4 (s.d. ± 5.1, range 49–68) years and 55.1 (s.d. ± 4.2, range 49–66) years in those taking HRT. Information regarding menopause length was known for 140 (76%) patients. Length of time since the menopause at time of surgery ranged from 3 to 300 months [median = 60, interquartile (IQ) range = 27–32 months] in the control group and 5–324 months (median = 65, IQ range = 36–96 months) in the HRT group. Data concerning duration of HRT use were known for 72 (97%) of the patients in the treatment group and ranged from 1 to 264 months (median = 36, IQ range = 12–66) months. Information regarding HRT dose was obtained for all treated patients. Daily oestradiol, oestradiol valerate or conjugated oestrogen doses, for patients taking either oestrogen alone or oestrogen and progestin combined therapy, ranged from 10 to 2000 µg. Parity status was known for 171 (92%) patients and 151 of these women had been through a full-term pregnancy.

A summary of the ranges and median Ki67 labelling index (LI) and PR LI from both treatment groups is presented in Table 1. Ki67 LI was measured in tissue from all the control patients (Figure 1A) and found to be unrelated to patient age (within the range of 49–68 years) ($n$ = 111, rho = 0.002, $P$ = 0.49). There was an insignificant trend towards a decrease in Ki67 LI with an increase in length of time since the menopause ($n$ = 86, rho = – 0.173, $P$ = 0.06). Tissue samples from 100 (90%) control patients were assayed for PR expression (Figure 1B). Expression was unaffected by patient age ($n$ = 100, rho = 0.07, $P$ = 0.25) and the length of time since menopause ($n$ = 78, rho = 0.112, $P$ = 0.16). In control patients normal breast tissue taken adjacent to a benign lesion showed no difference compared with tissue taken adjacent to a malignant lesion in either the Ki67 LI (benign lesions, $n$ = 31, median = 0.175, IQ range = 0.0–0.49; malignant lesions, $n$ = 80, median = 0.19, IQ range = 0.10–0.41) or PR LI (benign lesions, $n$ = 28, median = 4.6, IQ range = 0.71–8.02; malignant lesion, $n$ = 72, median = 4.75, IQ range = 1.45–8.77) (M–W $P$ = 0.4). Ki67 and PR LI were significantly correlated ($n$ = 100, rho = 0.294, $P$ = 0.001).

Tissue samples from all patients receiving HRT were assayed for Ki67 expression. There was no significant difference between Ki67 LI in patients receiving $E_2$ or $E_2$ + P HRT and the control group (K–W $P$ = 0.61) (Figure 1A). PR LIs in patients taking both types of HRT were significantly higher than the control group (K–W $P$ = 0.01) (Figure 1B), but did not differ significantly between the two types of HRT user. HRT dose and the duration of use did not correlate with any of the parameters measured.

**Figure 1** Ki67 (A) and PR (B) percentage LI of normal breast lobular epithelium from post-menopausal women. Patients either untreated (control) or on HRT (either $E_2$ alone or $E_2$+ P) at time of surgery. Median values indicated by line. *Statistically significantly different to control group (K-W $P$ = 0.01)

### DISCUSSION

In comparison with our studies of Ki67 expression in normal premenopausal breast (McMichael-Phillips et al, 1996), proliferation in the post-menopausal breast is markedly reduced. We previously demonstrated that Ki67 labelling averaged from 2.16% in the follicular phase to 12.60% in the luteal phase of the menstrual cycle using the same staining methods. Smaller studies have also shown a reduction in breast proliferation in post-menopausal women (Meyer and Connor, 1982; Walker et al, 1991). Oestradiol is one of the most potent stimulators of epithelial proliferation in the premenopausal breast and the severely reduced oestradiol levels present after the menopause presumably result in a lack of

© Cancer Research Campaign 1998

stimulation and the lowered proliferation rates that we have demonstrated. It is well documented that age also exerts a significant negative effect upon proliferation in premenopausal (Meyer 1977; Anderson et al. 1982; Potten et al. 1988) and post-menopausal (Meyer and Connor. 1982) breast epithelium. However, in this larger study, we were unable to find any influence of age upon any of the parameters measured in post-menopausal breast. Although the mechanism of the age effect remains unknown, our findings imply that the reduction in proliferation with increasing age may reach a plateau during the climacteric stage of menopause or at its commencement. There was a slight trend towards a reduction in proliferation with an increase in the number of months since the menopause; however, this remained insignificant.

We found that PR expression is maintained in the post-menopausal breast, although at a lower level than that in premenopausal women, in whom PR LI averages from 12.60% in the follicular phase to 16.45% in the luteal phase of the menstrual cycle (McMichael-Phillips et al. 1996). Oestradiol up-regulates PR expression in the epithelial elements of normal human breast xenografts (Laidlaw et al. 1995). Recent evidence suggests that PR expression is sensitive to low levels of oestradiol and higher levels are required to generate a proliferative response (Clarke et al. 1997). This could explain the reasonably high expression of PR in post-menopausal breast epithelium compared with the lowered rate of proliferation.

The use of exogenous hormones (e.g. the oral contraceptive) increases epithelial proliferation in normal premenopausal breast (Williams et al. 1991; Chang et al. 1995). Additionally, HRT increases proliferation of the endometrium, which is also an oestrogen-responsive tissue (Whitehead et al. 1981). The use of HRT ($E_2$ or $E_2$+ P) by our patients did not increase their breast epithelial proliferation index, suggesting that, postmenopausally, the breast may be less sensitive to oestrogen stimulation. A further explanation is that HRT failed to raise serum oestradiol to levels sufficiently high to stimulate proliferation, although levels were sufficient to treat menopausal symptoms. Serum samples from patients in this study were unavailable, but previous studies with a range of HRT preparations have been shown to raise serum oestradiol levels to those found in premenopausal women during the follicular phase of the menstrual cycle (Lind et al. 1979; Whittaker et al. 1980), and follicular phase oestradiol doses have been shown to increase significantly proliferation of normal premenopausal breast xenografts (Laidlaw et al. 1995). Although some patients in our study were receiving quite large levels of oestrogen, varying HRT doses did not relate to proliferation. A decline in premenopausal breast epithelial proliferation occurs with age, although the evidence that serum oestradiol levels also decline is lacking (Meyer 1977; Anderson et al. 1982; Potten et al. 1988), suggesting that sensitivity of the breast to oestradiol attenuates with age. This may result in the failure of low doses of oestrogen, in the form of HRT, to elicit a proliferative response. However, we did find that PR expression was higher in both types of HRT user compared with control subjects and there is evidence to suggest that the oestradiol dose required to up-regulate PR expression is lower than that required to induce proliferation within the human breast (Clarke et al. 1997). Our findings differ to those presented by Cline et al (1996) in which mammary proliferation was significantly increased in 'post-menopausal' macaque monkeys administered HRT. However, in that study, the reproductive history of each monkey was unknown and an early menopause was artificially induced. Ageing of the breast may have a role in attenuating oestrogen responsiveness and could account for the difference in response.

Endogenous and exogenous oestrogens are thought to contribute to breast cancer risk, primarily by increasing breast cell mitogenesis (Howell, 1989; Pike et al. 1993). Long-term HRT use has been shown in multiple studies to be associated with increased breast cancer risk (Brinton et al. 1981; Colditz et al. 1995; Beral et al. 1997) and it is thought this may be due to a sustained increase in breast proliferation over several years. Although duration of HRT use was considered in our analysis, we would not expect to pick up this effect using this type of study. We have provided evidence that HRT is oestrogenic upon post-menopausal breast tissue as PR expression is raised. We were unable to detect any significant increase in proliferation, but it is possible that patient numbers were insufficient to detect very small increases in normal breast proliferation. Some types of benign breast lesions are associated with an increased risk of subsequently developing cancer (McDivitt et al. 1992) and non-malignant lesions have been shown to express the oestrogen receptor (Jaquemier et al. 1982). This suggests that benign lesions could respond to exogenous oestrogen and HRT may increase breast cancer risk by promoting the development of these premalignant lesions, initiated earlier in the patient's life. In addition, anti-oestrogens are commonly used in the treatment of breast cancer and have been shown to decrease tumour cell proliferation *in vivo* (Clarke et al. 1993), suggesting that HRT could accelerate the growth and subsequent detection of early, preinvasive malignant lesions.

### ACKNOWLEDGEMENTS

We thank Shaheena Sarwar, Lesley Shaw, Vicki Harvey, Ashu Gandhi and the histology department of the PICR for technical assistance. We are also grateful to the Cancer Research Campaign for the use of their facilities and to the Ministry of Agriculture Fisheries and Food for providing the salary for DFH.

### REFERENCES

Anderson TJ, Ferguson DJP and Raab GM (1982) Cell turnover in the 'resting' human breast: influence of parity, contraceptive pill, age and laterality. *Br J Cancer* **46**: 376–382

Beral V, Bull D, Doll R, Key T, Peto R and Reeves G (1997) Breast cancer and hormone replacement therapy: collaborative reanalysis of data from 51 epidemiological studies of 52 705 women with breast cancer and 108 411 women without breast cancer. *Lancet* **350**: 1047–1059

Bergkvist L, Adami H, Persson I, Hoover R and Schairer C (1989) The risk of breast cancer after estrogen and estrogen–progestin replacement. *N Engl J Med* **321**: 293–297

Brinton LA, Hoover RN, Szklo M and Fraumeni JF (1981) Menopausal estrogen use and risk of breast cancer. *Cancer* **47**: 2517–2522

Bromley M, Rew D, Becciolini A, Balxi M, Chadwick C, Hewitt D, Li YQ and Potten CS (1996) A comparison of proliferation markers (Brd Urd, Ki-67, PCNA) determined at each cell position in the crypts of normal human colonic mucosa. *Eur J Histochem* **40**: 89–100

Chang K, Lee TYT, Linares-Cruz G, Fournier S and de Lignieres B (1995) Influences of percutaneous administration of estradiol and progesterone in human breast epithelial cell cycle in vivo. *Fertility Sterility* **63**: 785–791

Clarke RB, Laidlaw IJ, Jones LJ, Howell A and Anderson E (1993) Effect of tamoxifen on Ki67 labelling index in human breast tumours and its relationshiop to oestrogen and progesterone receptor status. *Br J Cancer* **67**: 606–611

Clarke RB, Howell A and Anderson E (1997) Estrogen sensitivity of normal human breast tissue *in vivo* and implanted into athymic nude mice: analysis of the relationship between estrogen-induced proliferation and progesterone receptor expression. *Breast Cancer Res Treat* **45**: 121–133

© Cancer Research Campaign 1998

Cline JM, Soderqvist G, Schoultz E, Skoog L and von Schoultz B (1996) Effects of hormone replacement therapy on the mammary gland of surgically postmenopausal cynomolgus macaques. *Am J Obstet Gynecol* **174**: 93–100

Colditz GA, Hankinson SE, Hunter DJ, Willett WC, Manson JE, Stampfer MJ, Hennekens C, Rosner B and Speizer FE (1995) The use of estrogens and progestins and the risk of breast cancer in postmenopausal women. *N Engl J Med* **332**: 1589–1593

Greendale GA and Judd HL (1993) The menopause: health implications and clinical management. *J Am Geriatr Soc* **41**: 426–436

Harding C, Knox WF, Faragher EB, Baildam A and Bundred NJ (1996) Hormone replacement therapy and tumour grade in breast cancer: prospective study in screening unit. *Br Med J* **312**: 1646–1647

Horwitz KB and McGuire WL (1978) Estrogen control of progesterone receptor in human breast cancer. *J Biol Chem* **253**: 2223–2228

Howell A (1989) Clinical evidence for the involvement of oestrogen in the development and progression of breast cancer. *Proc R Soc Edin* **95B**: 49–57

Howell A, Baildam A, Bundred N, Evans G and Anderson E (1995) 'Should I take HRT, doctor?' Hormone replacement therapy in women at increased risk of breast cancer and in survivors of the disease. *J Br Menopause Soc* **October**: 9–17

Jacquemier JD, Rolland PH, Vague D, Lietaud R, Spitalier JM and Martin PM (1982) Relationships between steroid receptor and epithelial cell proliferation in benign fibrocystic disease of the breast. *Cancer* **49**: 2534–2536

Jacquemier JD, Hassoun J, Torrente M and Martin P (1990) Distribution of estrogen and progesterone receptors in healthy tissue adjacent to breast lesions at various stages – immunohistochemical study of 107 cases. *Breast Cancer Res Treat* **15**: 109–117

Key TJA and Pike MC (1988) The role of oestrogens and progestagens in the epidemiology and prevention of breast cancer. *Eur J Cancer* **24**: 29–43

Laidlaw I, Anderson E, Clarke R, Potten CS and Howell A (1995) The proliferation of normal human breast tissue implanted into athymic nude mice is stimulated by oestrogen but not progesterone. *Endocrinology* **136**: 164–171

Lind T, Cameron EC, Hunter WM, Leon C, Moran PF, Oxley A, Gerrard J and Lind UCG (1979) A prospective, controlled trial of six forms of hormone replacement therapy given to postmenopausal women. *Br J Obstet Gynecol* **86,S3**: 1–29

McDivitt RW, Stevens JA, Lee NC, Wingo PA, Rubin GL and Gersell D (1992) Histologic types of benign breast disease and the risk for breast cancer. *Cancer* **69**: 1408–1414

McKinlay SM, Brambilla DJ, Posner JG (1992) The normal menopause transition. *Maturitas* **14**: 103–115

McMichael-Phillips DF, Harding C, Morton M, Roberts SA, Howell A, Potten CS and Bundred NJ (1996) The effects of soy supplementation on epithelial proliferation in the normal human breast. *Breast Cancer Res Treat* **41**: 263

Meyer JS (1977) Cell proliferation in normal human breast ducts, fibroadenomas, and other ductal hyperplasia measured by nuclear labelling with tritiated thymidine. *Human Pathol* **8**: 67–81

Meyer JS and Connor RE (1982) Cell proliferation in fibrocystic disease and postmenopausal breast ducts measured by thymidine labeling. *Cancer* **50**: 746–751

Moorhead T, Hannaford P and Warskyj M (1997) Prevalence and characteristics associated with use of hormone replacement therapy in Britain. *Br J Obstet Gynaecol* **104**: 290–297

Pike MC, Spicer DV, Dahmoush L and Press MF (1993) Estrogens, progestogens, normal breast cell proliferation, and breast cancer risk. *Epidemiol Rev* **15**: 17–35

Potten CS, Watson RJ, Williams GT, Tickle S, Roberts SA, Harries M and Howell A (1988) The effect of age and menstrual cycle upon proliferative activity of the normal human breast. *Br J Cancer* **58**: 631–634

Preston-Martin S, Pike MC, Ross RK, Jones PA and Henderson BE (1990) Increased cell division as a cause of human cancer. *Cancer Res* **50**: 7415–7421

Soules, MR and Bremner WJ (1982) The menopause and climacteric. *J Am Geriatrics Soc* **30**: 547–561

Sullivan JM and Fowlkes MD (1996) Estrogens, menopause and coronary heart disease. *Cardiol Clin* **14**: 105–116

Trichopoulos D, MacMahon B and Cole P (1972) Menopause and breast cancer risk. *J Natl Cancer Inst* **48**: 605–613

Walker KJ, Price-Thomas JM, Candlish W and Nicholson RI (1991) Influence of the antioestrogen tamoxifen on normal breast tissue. *Br J Cancer* **64**: 764–768

Wellings SR, Jensen HM and Marcum RG (1975) An atlas of subgross pathology of the human breast with special reference to possible precancerous lesions. *J Natl Cancer Inst* **55**: 231–243

Whitehead MI, Townsend PT, Pryse-Davies J, Ryder TA and King RJ (1981) Effects of estrogens and progestins on the biochemistry and morphology of the postmenopausal endometrium. *N Engl J Med* **305**: 1599–1605

Whittaker PG, Morgan MRA, Dean PDG, Cameron EHD, Lind T (1980) Serum equilin, oestrone and oestradiol levels in postmenopausal women receiving conjugated equine oestrogens ('Premarin'). *Lancet* **8158**: 14–15

Williams G, Anderson E, Howell A, Watson R, Coyne J, Roberts SA and Potten CS (1991) Oral contraceptive (OCP) use increases proliferation and decreases oestrogen receptor content of epithelial cells in the normal human breast. *Int J Cancer* **48**: 206–210

Yates RA, Dowsett M, Fisher GV, Selen A and Wyld PJ (1996) Arimidex (ZD1033): a selective, potent inhibitor of aromatase in postmenopausal female volunteers. *Br J Cancer* **73**: 543–548

© Cancer Research Campaign 1998